# Inactivation of *Tm6sf2*, a Gene Defective in Fatty Liver Disease, Impairs Lipidation but Not Secretion of Very Low Density Lipoproteins*

**DOI:** 10.1074/jbc.M116.719955

**Published:** 2016-03-24

**Authors:** Eriks Smagris, Shenise Gilyard, Soumik BasuRay, Jonathan C. Cohen, Helen H. Hobbs

**Affiliations:** From the Departments of ‡ Molecular Genetics and; § Internal Medicine and; the ¶ Howard Hughes Medical Institute, University of Texas Southwestern Medical Center, Dallas, Texas 75390

**Keywords:** cholesterol metabolism, lipid droplet, lipoprotein, liver injury, triacylglycerol

## Abstract

A missense mutation (E167K) in TM6SF2 (transmembrane 6 superfamily member 2), a polytopic protein of unknown function, is associated with the full spectrum of fatty liver disease. To investigate the role of TM6SF2 in hepatic triglyceride (TG) metabolism, we inactivated the gene in mice. Chronic inactivation of *Tm6sf2* in mice is associated with hepatic steatosis, hypocholesterolemia, and transaminitis, thus recapitulating the phenotype observed in humans. No dietary challenge was required to elicit the phenotype. Immunocytochemical and cell fractionation studies revealed that TM6SF2 was present in the endoplasmic reticulum and Golgi complex, whereas the excess neutral lipids in the *Tm6sf2*^−/−^ mice were located in lipid droplets. Plasma VLDL-TG levels were reduced in the KO animals due to a 3-fold decrease in VLDL-TG secretion rate without any associated reduction in hepatic apoB secretion. Both VLDL particle size and plasma cholesterol levels were significantly reduced in KO mice. Despite levels of TM6SF2 protein being 10-fold higher in the small intestine than in the liver, dietary lipid absorption was only modestly reduced in the KO mice. Our data, taken together, reveal that TM6SF2 is required to mobilize neutral lipids for VLDL assembly but is not required for secretion of apoB-containing lipoproteins. Despite TM6SF2 being located in the endoplasmic reticulum and Golgi complex, the lipids that accumulate in its absence reside in lipid droplets.

## Introduction

Uncertainty regarding the molecular basis of nonalcoholic fatty liver disease (NAFLD)^4^ has been a significant obstacle to the development of effective therapies. Obesity is the major risk factor for accumulation of excess hepatic fat, but liver fat content varies widely among obese individuals (1). Furthermore, many individuals with hepatic steatosis do not develop chronic liver disease (2). The reasons for these wide variations in susceptibility to NAFLD are not known. As a first step toward elucidating the pathophysiology of the disorder, we screened the Dallas Heart Study population for DNA sequence variations associated with liver fat content, as determined by proton magnetic resonance spectroscopy (3, 4). Analysis of ∼9,200 nonsynonymous sequence variants revealed a common missense substitution (rs738409, I148M) in PNPLA3 that is associated with the full spectrum of NAFLD, including liver fat content, steatohepatitis, cirrhosis, and hepatocellular carcinoma (5–9). The PNPLA3-148 M variant also increases the risk of cirrhosis in alcoholics (10–12), suggesting that alcoholic liver disease and NAFLD share common pathogenic elements. Subsequent genome-wide association studies revealed other common SNPs associated with fat accumulation in liver (7). More recently, an exome-wide association analysis of the Dallas Heart Study revealed a second missense variant (rs58542926) in *TM6SF2*, a gene of unknown function, that is associated with liver fat content, reduced plasma lipid levels, and increased levels of circulating liver enzymes (13, 56). The variant was an adenine-to-guanine substitution in coding nucleotide 499 that replaces glutamate at residue 167 with lysine (c.499A>G; p.Glu167Lys). Like PNPLA3-148M, the TM6SF2-167K variant is associated with not only NAFLD but also alcoholic fatty liver disease (14) and with the full spectrum of both disorders, extending from steatosis to cirrhosis (15–19).

To elucidate the biologic function of TM6SF2 and the pathogenic mechanisms of TM6SF2-associated liver disease, we generated mice in which expression of the gene was ablated by insertional mutagenesis using a gene trap vector. Here we show that chronic germ line inactivation of *Tm6sf2* leads to accumulation of TG in enterocytes as well as hepatocytes. TM6SF2 is expressed both in the ER and in the Golgi complex, and we provide evidence that the polytopic protein participates in lipidation of apoB-containing lipoproteins without interfering with secretion of the particles from the liver.

## Experimental Procedures

### 

#### 

##### Mice

Animals were maintained on a 12-h light/12-h dark cycle and fed *ad libitum* Teklad Rodent Diet 2016 (Harlan Teklad), a high fat diet (D12451; 45% kcal from lard; Research Diets), or a high sucrose diet (HSD) (number 901683; 74% kcal from sucrose; MP Biomedicals) with free access to water. All experimental protocols were approved by the University of Texas Southwestern Medical Center Institutional Animal Care and Research Committee. For the fasting-refeeding experiments, chow-fed male C57BL/6N mice (*n* = 4 mice/group, age 8 weeks) were fasted for 18 h (2:00 p.m. to 8:00 a.m.) and then refed with an HSD (number 901683, MP Biomedicals) for 6 h (8:00 a.m. to 2:00 p.m.) for 3 days. On the morning of the fourth day, mice in the fasted group were deprived of food for an additional 6 h (total: 24-h fast), and the refed group was given food (HSD) for 6 h. The two groups of mice were sacrificed simultaneously, and the livers were collected. For all other measurements, mice were fed *ad libitum* overnight, and food was withdrawn at 7:00 a.m. Blood and tissue samples were taken at 11:00 a.m., 4 h after food withdrawal.

Total body fat and lean body mass were determined by nuclear magnetic resonance imaging using a Minispec MQ10 analyzer (Bruker).

##### Generation and Validation of Tm6sf2 Knock-out Mice

**FIGURE 1.**
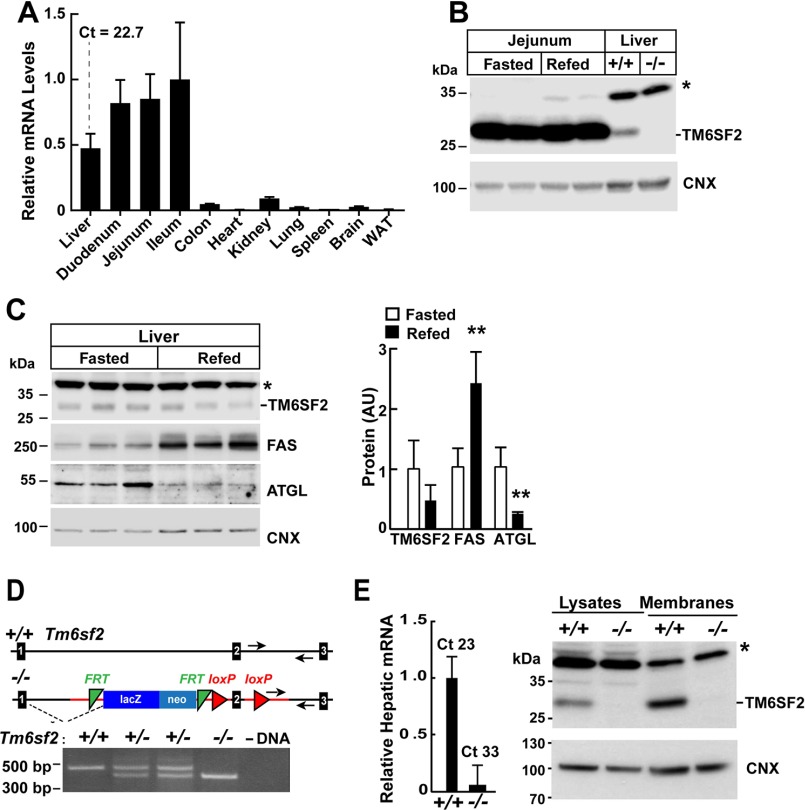
**Expression of *Tm6sf2* and generation of *Tm6sf2*^−/−^ mice.** *A*, total RNA was extracted from the indicated tissues of WT female mice (*n* = 3, age = 14 weeks) after a 4-h fast and subjected to quantitative real-time PCR as described under “Experimental Procedures.” The mean ± S.E. (*error bars*) levels of TM6SF2 transcript in each tissue are expressed relative to the expression level in the ileum, which was arbitrarily set to 1. *B*, regulation of TM6SF2 expression in response to fasting and refeeding. Mice were entrained to a synchronized feeding regimen for 3 days and then killed after a 24-h fast (*Fasted*) or after 18 h of fasting and 6 h of refeeding (*Refed*) (4 male mice, age = 8 weeks). Jejunal and liver proteins (60 μg/well) were size-fractionated by 10% SDS-PAGE, and immunoblotting analysis was performed using antibodies against TM6SF2 and calnexin. *C* (*left*), liver proteins (60 μg/well) from the experiment described in *B* were size-fractionated on an SDS-10% polyacrylamide gel. Fatty acid synthase (*FAS*) and adipose TG lipase (*ATGL*) and were used as positive controls for fasting and refeeding, and calnexin (*CNX*) was used as a loading control in this experiment. *C* (*right*), immunoblotting signals were quantified using a LI-COR Odyssey Fc imager. *D*, *Tm6sf2*^−/−^ mice were generated as described under “Experimental Procedures.” Genotyping was performed by PCR using oligonucleotides (*arrows*) to amplify a 470-bp (WT) or 400-bp (KO) fragment from genomic DNA. *E* (*left*), RNA was isolated from livers of male WT and KO mice (*n* = 3 male mice/group, 14 weeks old), and TM6SF2 expression was determined by quantitative real-time PCR as described under “Experimental Procedures.” The level of TM6SF2 transcript in WT mice was arbitrarily set to 1. *E* (*right*), immunoblotting analysis of hepatic TM6SF2 in 7-week-old female WT and KO mice. Liver lysates and membranes were prepared as described under “Experimental Procedures.” Aliquots of each fraction (50 μg) were size-fractionated by SDS-PAGE, and immunoblotting was performed using a rabbit anti-mouse TM6SF2 polyclonal antibody (1:1000) as described under “Experimental Procedures.” Calnexin served as a loading control for the experiment. *, nonspecific band. All experiments were repeated at least once, and the results were similar. *Ct*, cycle threshold. Values are means ± S.E. **, *p* < 0.01. *AU*, arbitrary units.

Mouse embryonic stem cells in which one *Tm6sf2* allele was inactivated (Fig. 1*D*) were purchased from the Knock-out Mouse Project (Davis, CA). The cells were injected into C57BL/6N (B6N-Tyrc-Brd/BrdCrCrl, albino, Charles River Laboratories) early stage mouse embryos, and the embryos were implanted into the uteri of C57BL/6N albino female mice. Chimeric males generated from these embryos were selected, and those with the greatest proportion of mutant cells (assessed by coat color) were bred with C57BL/6N albino females (Charles River Laboratories). Black offspring were genotyped and back-crossed with C57BL/6N females. Offspring of heterozygous (*Tm6sf2*^+/−^) matings were used for all experiments described in this paper. DNA (∼100 ng) extracted from the tails of mice was PCR-amplified using two forward oligonucleotides (5′-taattatgtctttctcccctatgccc-3′ (WT) and 5′-ttatacgaagttatggtctgagctcg-3′ (KO)) and a reverse primer (5′-ggcagaggcaggcaagtttc-3′). PCR products were size-fractionated on 2% agarose gels and visualized with ethidium bromide.

##### RNA Expression

mRNA levels were determined by real-time PCR as described (20). Mouse 36B4 was used as an internal control.

##### Liver and Plasma Chemistries

Lipids were extracted from pieces of liver (95–125 mg) using the method of Folch and Lees (21). Tissue levels of TG, cholesterol, cholesteryl ester, free cholesterol, and phosphatidylcholine were measured using enzymatic assays (Infinity, Thermo Electron Corp., and Wako Inc.) and normalized to sample weight. Serum levels of alanine transaminase, aspartate aminotransferase, TG, cholesterol, albumin, and glucose were measured using the Vitros 250 system (GMI Inc.). Serum levels of nonesterified fatty acids were measured by enzymatic assay using a commercial reagent (Wako Inc.). ELISAs were used to quantify serum insulin (Crystal Chem Inc.) and plasma PCSK9 levels. For the PCSK9 assays, LumiNunc Maxisorp assay plates (NalgeNunc) were coated with polyclonal rabbit anti-mouse PCSK9 antibody (clone IgG-551C, Jay Horton, University of Texas Southwestern Medical Center) diluted to 5 μg/ml in 100 μl of Buffer A (100 mm NaCl, 20 mm Na_3_PO_4_, pH 7.5). After an overnight incubation at 4 °C, plates were washed three times with 350 μl of PBST (PBS with Tween 20 (0.05%), pH 7.4) and then incubated in 150 μl of 0.5% BSA in Buffer A for 1 h at room temperature. All subsequent plate washes were conducted on a BioTek ELv405 plate washer. Standards and antibodies were diluted in Buffer A plus 0.5% BSA. Duplicate plasma samples (1 μl) were applied to each well. A standard curve was constructed using purified mouse PCSK9 serially diluted to final concentrations of 0.39–50 ng/ml. Plates were shaken at 37 °C for 2.5 h, and then the samples were washed before adding 100 μl of rabbit anti-mouse PCSK9 polyclonal antibody (IgG-552C, biotinylated with EZ-Link Sulfo-NHS-Biotin kit, Pierce). After 2 h of incubation at room temperature, the plate was washed again. A total of 100 μl of avidin-HRP (1:40,000; Pierce) was added and incubated for 1 h at room temperature. After a final wash, 100 μl of SuperSignal ELISA Pico Substrate (Pierce) was added for 1 min. Luminescence was quantified using a ThermoFisher Luminex luminometer. Linear regression analysis of the standard curve was used to determine concentrations of PCSK9.

To measure plasma lipid and lipoprotein levels, blood was collected from mice fasted for 4 h, and plasma from 4–5 mice was pooled (total volume 400 μl) and size-fractionated on a fast performance liquid chromatography (FPLC) Superose 6 column (GE Healthcare). A total of 42 fractions (300 μl each) were collected, and cholesterol and TG content of each fraction were measured using enzymatic assays (Infinity, Thermo Scientific).

To analyze the fatty acid composition of different lipid classes, pieces of liver (50–60 mg) were homogenized in 5 ml of chloroform/methanol (2:1, v/v) and 1 ml of NaCl. Homogenized samples were centrifuged at 4,000 × *g* for 10 min at room temperature, and the lower phases were collected. One-third of the extract was dried under N_2_, and the residue was resuspended in 100 μl of chloroform. Triglycerides, phospholipid (phosphatidylcholine), cholesteryl esters, and nonesterified fatty acids were separated by thin layer chromatography on silica plates using hexane/diethyl ether/acetic acid (120, 30, and 3 ml, respectively) as the mobile phase. Bands corresponding to each lipid class were visualized using iodine vapor, excised, and placed in 2 ml of methanol/benzene (4:1). Acetyl chloride (200 μl) was added as the sample was vortexed. The sample was incubated at 100 °C for 1 h and then allowed to cool to room temperature. Once at room temperature, 5 ml of 6% aqueous K_2_CO_3_ was added slowly, followed by the addition of 1.5 ml of benzene. Samples were mixed and centrifuged at 4,000 × *g* for 10 min. The upper phase was removed, dried under N_2_, and reconstituted in 150 μl of hexane. Fatty acids were measured by gas chromatography (GC) using a Hewlett Packard 6890 series GC system. The identities of the fatty acids were determined by comparing the retention times with fatty acid standards (GLC-744, NU-Chek Prep) using pentadecanoic acid (C15:0) as an internal standard. Fatty acid concentrations were calculated based on the area of the C15:0 peak. Sterols were extracted from bile collected from the gall bladders of mice after a 4-h fast and measured using GC-MS. Eluted sterols were identified and quantified using electron ionization-MS in single-ion-monitoring mode, as described previously (22). Individual sterols were identified using a pattern of three ions. The sterol levels were quantified using ions with the following *m*/*z*: cholesterol, 458; campesterol, 382; sitosterol, 396; stigmasterol, 484.

##### Tissue Morphology

For Oil Red O staining, livers were fixed in 4% paraformaldehyde for 24 h and then equilibrated in 10% and then 18% sucrose (all in PBS) for 24 h each at 4 °C. Tissue was cryosectioned, brought to room temperature, and air-dried for 2 h and then fixed in methanol-free paraformaldehyde (4%). Slides were washed three times with H_2_O and then incubated for 10 min in 0.18% Oil Red O (Sigma) prepared in 60% isopropyl alcohol. Slides were washed five times in H_2_O. Nuclei were counterstained with hematoxylin, and the coverslips were affixed with aqueous mounting medium (Vector Laboratories). Sections were visualized using a Leica microscope (DM2000) at ×20, ×40, and ×63 magnification. The numbers and sizes of lipid droplets (original magnification ×63) were determined in five randomly selected images from each slide (*n* = 4 mice/group) using ImageJ (National Institutes of Health).

##### Electron Microscopy

To image very low density lipoprotein (VLDL) using electron microscopy, 400 μl of plasma was obtained from WT and KO mice after a 4-h fast. Plasma was placed at the bottom of an ultracentrifuge tube (1 ml) and overlaid with 600 μl of KBr (1.006 g/ml) solution. Samples were centrifuged at 434,000 × *g* for 2 h at 16 °C in a S120-AT2 rotor. A total of 200 μl from the top of tube was collected (VLDL fraction). A 5-μl aliquot of the VLDL fraction was applied to a carbon-coated copper grid and stained with 2.0% uranyl acetate for 15 min (23). Grids were visualized with a Tecnai G2 Spirit transmission electron microscope (FEI). Images of VLDL were recorded on a Gatan 2,000 × 2,000 CCD camera, and the size distribution of the particles was determined using ImageJ (10 random images/group).

##### Western Blotting

Liver tissue (50–100 mg) was homogenized in RIPA buffer (150 mm NaCl, 1.0% IGEPAL CA-630, 0.5% sodium deoxycholate, 0.1% SDS, and 50 mm Tris, pH 8.0, supplemented with protease inhibitors (Protease Inhibitor Mixture, Roche Applied Science)). Lysates were cleared by centrifugation (12,000 × *g*) for 15 min at 4 °C, mixed with 2× Western blot Laemmli sample buffer (65.8 mm Tris, pH 6.8, 2.1% SDS, 26.3% (w/v) glycerol, 0.01% bromphenol blue, 5% (v/v) β-mercaptoethanol), and incubated at 37°C for 1 h. Samples were size-fractionated by 10% SDS-PAGE, and proteins were transferred to a PVDF membrane (Immobilin-FL, Millipore). Membranes were incubated overnight at 4 °C with antibodies against the following proteins (source, catalogue number, and dilution of the antibodies are given in parentheses): calnexin (Enzo, ADI-SPA-860-F; 1:5,000), PLIN2 (perlipin 2) (Fitzgerald Industries International, 20R-AP002; 1:5,000), lactate dehydrogenase (Abcam, ab134187; 1:1,000), PNPLA3 (R&D Systems, AF5179; 1:1,000), FASN (320D, rabbit polyclonal anti-mouse antibody, gift from Jay Horton; 1:5,000), adipocyte TG lipase (Cell Signaling, 2439S; 1:1,000), SAR1b (Abcam, ab155278; 1:5,000), microsomal triglyceride transfer protein (MTTP) (BD Biosciences, 612022; 1:5,000), calnexin N terminus (Novus, NB100-1965; 1:1,000), BiP (Cell Signaling, 3183S; 1:1,000), GOS28 (Abcam, ab82961; 1:1,000), phospholipid transfer protein (Novus, NB400-106; 1:500), LSD1 (Cell Signaling, 2184; 1:1,000), and apoB (Abcam, ab20737, 1:1000). A polyclonal rabbit anti-mouse anti-TM6SF2 antibody (792D) and mouse mAbs (clones 8B3-B7 and 8B3-E1) were raised against a keyhole limpet hemocyanin-conjugated peptide corresponding to the C-terminal 15 amino acids of mouse TM6SF2 (CALPSSPQDQDKKQQ obtained from GenScript). We used a goat anti-mouse IgG, Fcγ fragment-specific antibody (Jackson ImmunoResearch Inc., 115-035-071; 1:3000) to detect the primary anti-mouse TM6SF2 antibody.

To detect apoB, liver lysates were prepared by homogenizing tissue in modified RIPA buffer (150 mm NaCl, 1.0% IGEPAL CA-630, 0.5% sodium deoxycholate, 1% SDS, 1 m urea, and 50 mm Tris, pH 8.0) supplemented with protease inhibitors. Lysates were size-fractionated by 5% SDS-PAGE and then transferred overnight to a Hybond-C membrane (GE Healthcare) at 0.1 A at 4 °C overnight. Membranes were incubated with an apoB antibody overnight at 4 °C. ApoB was detected in mouse plasma by immunoblotting. Plasma (10 μl) was added to 50 μl of 2× Laemmli sample buffer and 40 μl of RIPA buffer, and 2 μl of the mixture was size-fractionated on 4–12% BisTris gels (Bio-Rad).

To assess the processing of SREBP-1c (sterol regulatory element-binding protein-1c), mice were metabolically synchronized for 3 days (fasting from 8:00 a.m. until 8:00 p.m.; refeeding 8:00 p.m. until 8:00 a.m.) and sacrificed at the end of the last refeeding cycle. Livers were collected and stored at −80°C. A total of 100 mg of liver was homogenized on ice in 1 ml of homogenization buffer (20 mm Tris-Cl, pH 7.4, 2 mm MgCl_2_, 0.25 m sucrose, 10 mm EDTA, 10 mm EGTA, 5 mm DTT, and 1 mm PMSF) supplemented with 5× protease inhibitors using a T10 mechanical homogenizer (IKA) set on maximum speed. Lysates were centrifuged for 5 min at 3,000 × *g* at 4 °C, and 800 μl of supernatant was collected (the postnuclear supernatant). The remaining pellet and supernatant were maintained at 4 °C (the nuclear fraction). The postnuclear supernatant was centrifuged at 100,000 × *g* for 30 min (47,100 rpm; Sorvall S120AT2 rotor), and the pellet was collected (membrane fraction). The membrane fraction was dissolved in 400 μl of RIPA buffer, and the protein concentration was determined using a BCA kit (Pierce). The remaining supernatant was aspirated from the nuclear fraction, and the pellet was resuspended in 1 ml of homogenization buffer and centrifuged for 5 min at 1,000 × *g* at 4 °C. The supernatant was removed, and the pellet was resuspended in 300 μl of Buffer B (20 mm HEPES, pH 7.6, 2.5% glycerol (v/v), 0.42 m NaCl, 1.5 mm MgCl_2_, 1 mm EDTA, 1 mm EGTA supplemented with 5× protease inhibitor). The resuspended nuclei were rocked for 45 min at 4 °C and then centrifuged at 100,000 × *g* for 30 min (47,100 rpm, Sorvall S120AT2 rotor). The supernatant (nuclear extract) was collected, and the protein concentration was determined. A total 20 μg of protein was mixed with 6XSDS sample buffer (0.375 m Tris, pH 6.8, 12% SDS, 60% glycerol (v/v), 0.6 m DTT, 0.06% bromphenol blue) and heated at 95 °C for 8 min. Equal volumes of nuclear and membrane fractions from each group (*n* = 4) were pooled, and 40 μg of protein was loaded onto an SDS-10% polyacrylamide gel. The blot was incubated overnight at 4 °C with rabbit anti-mouse SREBP-1c antibody that was developed in the Department of Molecular Genetics (University of Texas Southwestern). After signal detection, membranes were stripped (Restore Western blot stripping buffer, Thermo Scientific) and reblotted with antibodies against calnexin and LSD1 to determine the purity and loading consistency of each fraction. The ECL signal was detected and quantified using a LI-COR Biosciences Odyssey Fc imager.

##### VLDL and ApoB Secretion

To measure rates of VLDL-TG secretion, five female mice (10 weeks old) of each genotype were fasted for 4 h at the beginning of the light cycle. A bolus of Triton WR-1339 (Tyloxapol, Sigma-Aldrich) (500 mg/kg) was given via the tail vein (24). Blood samples (∼40 μl) were collected from the tail vein in EDTA-coated tubes (Microvette 500 KE, Sartedt) before and 30, 60, 90, and 120 min after the injection of detergent.

ApoB secretion rates were measured exactly as described previously (25, 26). Briefly, each mouse was injected with 200 μCi of [^35^S]methionine (1,175 Ci/mmol) (PerkinElmer Life Sciences) and Triton WR-1339 (500 mg/kg). Blood was collected from the tail vein into aprotinin-containing EDTA-coated tubes, before and 45 and 90 min after the injection. Plasma from the 90 min time point was isolated, diluted 1:20 in PBS containing protease inhibitors, and incubated with deoxycholate (100 μl of 0.15% solution) for 10 min at room temperature. Trichloroacetic acid (TCA) (50 μl) was added, and the mixture was incubated for 30 min on ice and then centrifuged at 10,000 × *g* for 15 min at 4 °C. The supernatant was removed, and the pellet was resuspended, washed twice in ice-cold acetone, and centrifuged at 10,000 × *g* for 15 min at 4 °C. Pellets were air-dried, dissolved in buffer containing 2% SDS and 1 m urea, and heated to 37 °C for 1 h. Samples were mixed with 6× SDS sample buffer, heated for 5 min at 95 °C, and size-fractionated by SDS 5%-PAGE. Gels were dried and exposed to x-ray film (BIOMAX XAR) for 4 days at −80 °C. Films were scanned using an HP Scanjet 5590 and quantified using LI-COR Biosciences Image Studio software.

##### Fat Tolerance and Lipid Absorption Test

Chow-fed mice were fasted for 16 h and then gavaged with 10 μl of corn oil/g of mouse body weight. Blood samples were collected from the tail vein at the indicated times, and plasma TG levels were determined using a colorimetric assay (Infinity, Thermo Scientific).

To measure TG absorption, mice were fasted for 16 h prior to injection of Triton WR-1339 (500 mg/kg) into the tail vein. After 30 min, the mice were gavaged with 50 μl of corn oil (Sigma, C8267) supplemented with 5 μCi of 9,10-^3^H-labeled oleic acid (54.5 Ci/mmol; PerkinElmer Life Sciences) and 1 μCi of ^14^C-labeled cholesterol (50.8 mCi/mmol; PerkinElmer Life Sciences). Blood was collected from the tail vein before and 1, 2, 3, 4, and 7 h after Triton WR-1339 injection, and plasma levels of TG were measured by an enzymatic assay. Plasma samples (10 μl) were subjected to scintillation counting to quantitate the radioactivity.

##### Subcellular Fractionation

Female C57BL/6N mice (age 12 weeks) were fed an HSD for 2 weeks. Mice were fasted for 18 h and refed with the HSD for 6 h prior to being killed. Lipid droplets and lipid droplet proteins were isolated from the liver as described previously (27). Livers were maintained at 4 °C on ice in 10 ml of Buffer C (250 mm sucrose, 20 mm Tricine, pH 7.8) plus protease inhibitors and then homogenized using a Dounce type glass homogenizer. Lysates were centrifuged at 17,738 × *g*, and the lipid droplet fractions were collected from the tops of the tubes. Pellets and supernatants were resuspended in 10 ml of Buffer C and centrifuged at 1,000 × *g* for 10 min at 4 °C. The supernatant (postnuclear fraction) was collected, and a 1-ml aliquot was diluted in 9 ml of buffer C and then subjected to ultracentrifugation in a TH-641 swinging bucket rotor at 100,000 × *g* for 45 min at 4 °C. The supernatants (cytosol fraction) were collected, and the pellets were washed with PBS and then solubilized in 0.5 ml of RIPA buffer. The cytosol was concentrated to a volume of 200 μl using Amicon Ultra-15 centrifuge filters (Millipore, UFC901096) and then diluted with RIPA buffer. Laemmli sample buffer 2× (Bio-Rad) was added, and the samples were heated at 37 °C for 1 h. Proteins from the membrane and cytosolic fractions (50 μg each) and one-tenth of the lipid droplet proteins were size-fractionated by 10% SDS-PAGE and immunoblotted overnight at 4°C with antibodies to calnexin, lactate dehydrogenase, perilipin 2, PNPLA3, and a mouse anti-TM6SF2 mAb (clone 8B3-B7, 5 μg/ml).

ER and Golgi fractions were isolated by affinity chromatography as described (28, 29). Magnetic beads (2 ml of Dynabeads M-450 Epoxy, Invitrogen) were washed five times with ice-cold buffer D (0.1 m phosphate buffer, pH 7.9) and pelleted with a Dynal magnetic bead separator (Invitrogen). Goat anti-rabbit IgG (500 mg; Jackson ImmunoResearch Inc.) in 1.5 ml of Buffer D was added to the washed beads and slowly rotated for 72 h at 4 °C. Beads were washed again five times with ice-cold Buffer D and divided into two equal portions. The samples were then resuspended in 1.5 ml of Buffer D. To capture the ER, one-half of the beads were incubated with rabbit anti-calnexin antibody (90 μg) (Enzo Life Sciences, ADI-SPA-860-F). To capture the Golgi, the remaining portion of washed beads was added to 20 μg of rabbit anti-GM130 antibody (GeneTex Inc., GTX61445) and rotated for 24 h at 4 °C. Beads were then washed five times with Buffer D and again divided into two parts (for WT and KO samples) in preparation for incubation with mouse liver microsomes.

To prepare liver microsomes, mice were entrained to a synchronized regimen for 3 days by fasting from 8:00 a.m. to 8:00 p.m. and refeeding with chow overnight. At the end of the final 12-h refeeding period, the mice were killed, and their livers were removed and kept on ice throughout the procedure. Pieces of liver (600 mg) were disrupted in 3.0 ml of Buffer E (10 mm HEPES, pH 7.4, 10 mm KCl, 1.5 mm MgCl_2_, 5 mm sodium EDTA, 5 mm sodium EGTA, 250 mm sucrose, supplemented by protease inhibitors) using a T10 mechanical homogenizer (IKA) at maximal speed and then passed through a syringe 20 times with a 20-gauge needle. The homogenate was centrifuged at 2,000 × *g* for 10 min at 4 °C. The postnuclear supernatant was collected and centrifuged (21,000 × *g*) at 4 °C for 30 min. One-third of the pellet was resuspended in RIPA buffer. The remainder was resuspended in buffer containing 1.4 ml of Buffer D and 0.7 ml of Buffer E plus protease inhibitors. A total of 1.0 ml of the mixture was slowly rotated in the presence of antibody-coated beads for 24 h at 4°C. The supernatant was then removed, and the beads were washed 10 times with BSA (5%) in PBS with protease inhibitors and then another 10 times in 0.1% BSA in PBS and finally 10 times with PBS alone. Washed beads were incubated in 200 μl of buffer containing 2% SDS, 1 m urea plus 50 μl of 6× SDS sample buffer. Samples were heated for 1 h at 37 °C, and then the supernatants were collected and subjected to 10% SDS-PAGE.

##### Immunofluorescence Microscopy

Mice were anesthetized with isoflurane, and a catheter was inserted through the inferior vena cava. The liver was perfused (3 ml/min) with 30 ml of liver perfusion medium (Invitrogen, 17701-38) and then with 30 ml of liver digestion medium (Invitrogen, 17701-34). Livers were excised and placed in 100-mm plastic dishes containing 20 ml of liver digestion medium preheated to 37 °C. The surface capsule of the liver was removed, and the hepatocytes were gently dispersed. Cells were filtered through a 100 μm nylon mesh cell strainer (Falcon, Corning) and then transferred to a conical tube containing 20 ml of washing medium (DMEM plus glucose (1,000 mg/liter), FCS (5%) plus 50 units/ml penicillin, and 50 mg/ml streptomycin) and centrifuged at 40 × *g* at 4 °C for 5 min. Supernatants were discarded, and cells were resuspended in washing medium. Cells were seeded at a density of 0.5 × 10^6^ cells/well on 6-well plates containing coverslips (Fisher) precoated with rat tail collagen (Enzo Lifesciences). After 3 h, the cells were washed with PBS, fixed with 4% paraformaldehyde in PBS, and quenched in 50 mm ammonium chloride for 15 min. Cells were then permeabilized for 2 min in 0.1% (v/v) Triton X-100 in blocking buffer (0.4% fish skin gelatin (Sigma, G7765) in PBS). Cells were incubated with primary antibody in blocking buffer for 1 h and with secondary antibody for 30 min at room temperature. Coverslips were mounted on slides using Vectashield mounting medium with DAPI (Vector Laboratories). Slides were viewed under a Leica TCS-SP5 broadband confocal microscope using a ×63/1.30 numeric aperture oil immersion objective. All images were exported as tiff files and compiled using ImageJ software. TM6SF2 was detected using a mouse mAb (clone 8B3-B7) generated as described above. The following commercial antibodies were used: rabbit polyclonal antibodies against giantin and calnexin from Abcam, rabbit polyclonal antibody against PLIN2 from Thermo Scientific, and rabbit mAb against RCAS1 from Cell Signaling Technology. BODIPY 493/503 dye was purchased from Life Technologies, Inc. All other reagents were purchased from Sigma-Aldrich.

## Results

### 

#### 

##### TM6SF2 Expression Is Not Regulated by Food Intake

To determine the tissue distribution of TM6SF2 expression in mice, we measured levels of TM6SF2 mRNA in tissues collected from female WT C57Bl/6N mice after a 4-h fast (Fig. 1*A*). TM6SF2 expression was highest in liver and small intestine. To determine whether food intake alters TM6SF2 expression, we collected the jejunum and liver from mice fasted for 24 h and from mice fasted for 18 h and then refed for 6 h. TM6SF2 protein levels were ∼10-fold higher in jejunum than in liver (Fig. 1*B*) and were not significantly affected by fasting or refeeding in either tissue (Fig. 1, *B* and *C*). Levels of TM6SF2 mRNA were slightly increased in response to refeeding in one experiment, but this change was not observed consistently (data not shown) and was not reflected in the levels of TM6SF2 protein (Fig. 1*C*). Expression levels of fatty acid synthase (FAS) and adipocyte TG lipase (ATGL) were assayed as positive controls for the feeding protocol and showed the expected changes (Fig. 1*C*).

##### Establishing TM6SF2-deficient Mice

To determine the effect of stably inactivating *Tm6sf2* in all tissues, we established *Tm6sf2*^−/−^ mice from two independent embryonal stem cell lines. A map of the genetically modified locus is shown in Fig. 1*D*. All mice used in the experiments described in this paper were offspring from matings between heterozygotes derived from one of these lines (EPD0097_3_F02), and the major findings were validated in mice derived from the second line (EPD0097_3_E02) (data not shown).

Livers of *Tm6sf2*^−/−^ mice expressed almost no TM6SF2 mRNA (Fig. 1*E*, *left*) and had no detectable TM6SF2 protein (Fig. 1*E*, *right*). Both male and female KO mice were fertile. Litters from matings between heterozygous mice (*Tm6sf2*^+/−^) were comparable with those obtained from WT mice of the same strain (5.3 ± 2.3 *versus* 5.0 ± 2.5 pups/litter). The sex ratios were similar among WT, *Tm6sf2*^+/−^, and *Tm6sf2*^−/−^ offspring. Although fewer KO offspring were obtained from heterozygous parents than expected by chance, the difference did not reach statistical significance (*p* = 0.28) (data not shown).

**FIGURE 2.**
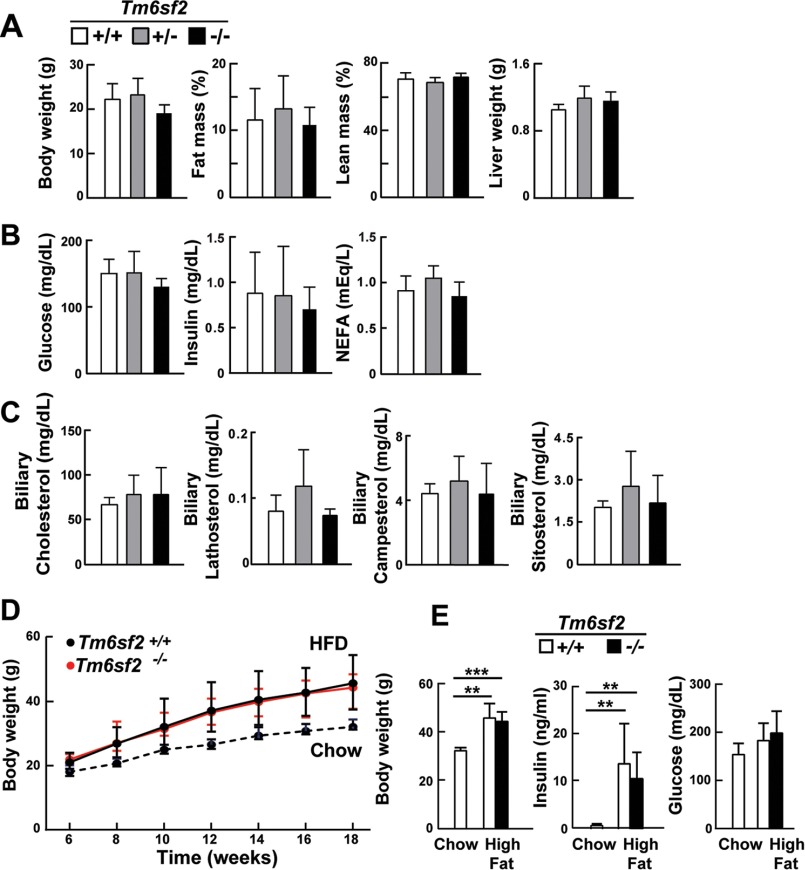
**Body composition (*A*), plasma chemistries (*B*), biliary sterols (*C*), and responses to a high fat diet (*D* and *E*) in WT and *Tm6sf2*^−/−^ mice.** *A*, fat mass and lean body mass of chow-fed male mice (9 weeks old, *n* = 5) were measured by NMR using a Minispec analyzer (Bruker). Body and liver weights were measured after the mice were killed at 13 weeks of age. *B*, plasma glucose, insulin, and non-esterified fatty acids (*NEFA*) were measured after a 4-h fast in the same mice at 11 weeks, as described under “Experimental Procedures.” *C*, sterols were extracted from gall bladder bile of male mice described in *A* and measured by GC-MS as described previously (22). *D*, male WT and KO mice (age = 6 weeks, *n* = 5 mice/group) were switched from a chow to a high fat diet at 6 weeks of age and fed with high fat diet for 12 weeks. A similar number of WT mice were simultaneously fed a chow diet. Body weights were measured at 2-week intervals, and final body weights plus plasma glucose and insulin levels (*E*) were measured at 18 weeks after a 4-h fast. Values are means ± S.E. (*error bars*). **, *p* < 0.01; ***, *p* < 0.001.

Inactivation of *Tm6sf2* did not adversely affect postnatal development: body weights, fat mass, lean mass, and liver weights were similar in 13-week-old chow-fed male KO and WT mice (Fig. 2*A*). Similar findings were seen in female mice and in mice derived from the second embryonic stem cell line (data not shown). Serum glucose, insulin, and nonesterified fatty acid levels after a 4-h fast (Fig. 2*B*) and serum glucose levels after a 24-h fast (92 ± 10 *versus* 90 ± 5 mg/dl, *p* = 0.86; *n* = 4 male mice) (data not shown) did not differ between the strains. Biliary sterol levels were similar in KO and WT animals (Fig. 2*C*). As expected, body weights and fasting serum insulin levels were increased after the mice consumed a high fat diet for 12 weeks, but the increases were similar in WT and KO animals (Fig. 2, *D* and *E*).

##### Chow-fed Tm6sf2^−/−^ Mice Develop Hepatic Steatosis

**FIGURE 3.**
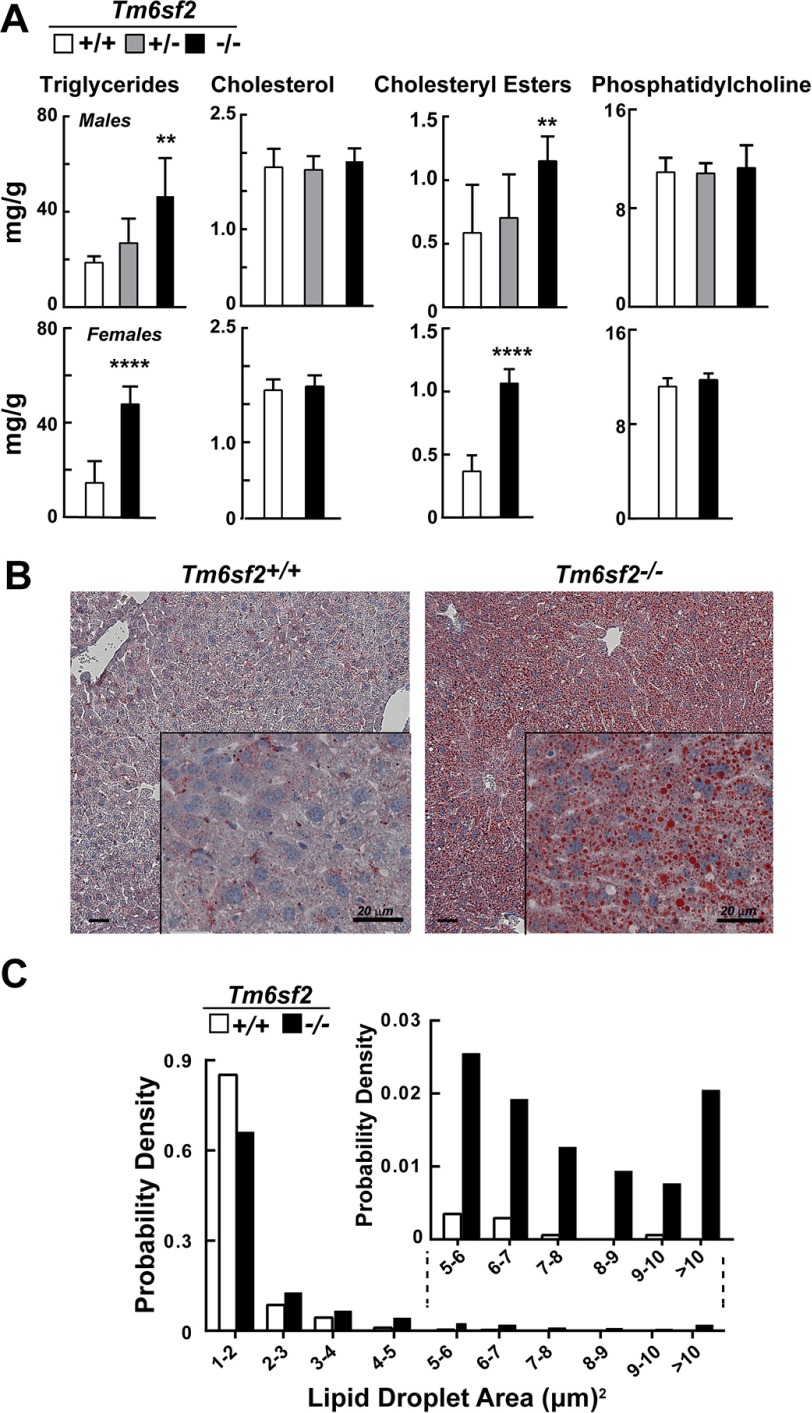
**Hepatic lipid content and LD size distribution in *Tm6sf2*^−/−^ mice.** *A*, hepatic lipid levels were measured in 13-week-old chow-fed male mice (5 mice/group) (*top*) and 11-week-old female mice (6 mice/group) (*bottom*) using enzymatic assays. *Bars*, means ± S.E. (*error bars*). *B*, liver sections from WT and *Tm6sf2*^−/−^ male mice from *panel A* were stained with Oil Red O and hematoxylin and visualized using a Leica microscope (DM2000) at ×10 and ×40 (*inset*) magnification. *C*, size distribution of male hepatic LDs in WT and KO mice. LD sizes were determined from images of Oil Red O-stained liver sections using ImageJ software as described under “Experimental Procedures.” The experiment was repeated twice, and the results were similar. **, *p* < 0.01; ****, *p* < 0.0001.

Levels of TG and cholesteryl esters were increased in livers of chow-fed *Tm6sf2*^−/−^ male mice compared with their WT littermates (Fig. 3*A*, *top*). Heterozygous mice had levels of TG and cholesteryl esters that were intermediate between those of WT and homozygous KO animals, which is consistent with the inactivating allele having a co-dominant effect. More pronounced increases in neutral lipids were seen in chow-fed female *Tm6sf2*^−/−^ mice (Fig. 3*A*, *bottom*). Hepatic levels of free cholesterol and phosphatidylcholine were similar in WT and KO animals of both sexes.

**FIGURE 4.**
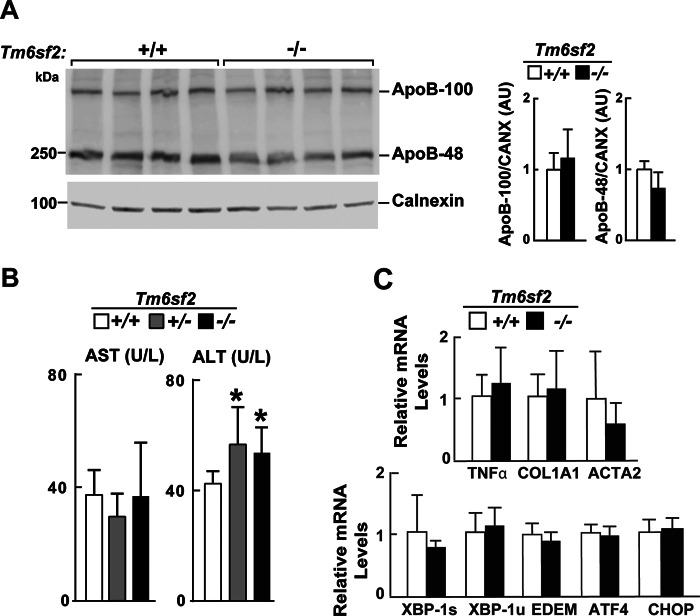
**Hepatic apoB levels (*A*), liver function tests (*B*), and relative hepatic levels of selected transcripts encoding proteins involved in activation of fibrosis and ER stress (*C*) in male WT and Tm6sf2^−/−^ mice described in Fig. 3*A*.** *A*, immunoblotting analysis was performed on liver protein (50 μg) using a rabbit anti-mouse apoB polyclonal antibody (1:1,000; Abcam) and ECL (SuperSignal West Pico Kit, Thermo Scientific). The ECL signal was visualized using a LI-COR imager (Odyssey Fc imager) and analyzed using LI-COR Image Studio software. *B*, plasma levels of aspartate aminotransferase (*AST*) and alanine aminotransferase (*ALT*) in chow-fed male mice. *C*, RNA levels were detected using quantitative real-time PCR (*qRT-PCR*), normalized to levels of 36B4 and expressed relative to the levels in the WT animals (*n* = 5). *COL1A1*, collagen alpha-1(I) chain; *ACTA2*, actin, aortic smooth muscle; *XBP1s/u*, X-box-binding protein 1 spliced/unspliced; *ATF4*, activating transcription factor 4; *EDEM*, ER degradation enhancer, mannosidase α-like 1; *CHOP(DDIT3)*, C/EBP-homologous protein. Values are means ± S.E. (*error bars*). *, *p* < 0.05. *AU*, arbitrary units.

Oil Red O staining of liver sections (Fig. 3*B*) revealed an increase in the number (417 *versus* 47 droplets/8,404-μm^2^ slide field) and the median size (2.2 *versus* 1.6 μm^2^, *p* = 0.023) of lipid droplets (LDs) in the KO animals (Fig. 3*C*). To limit artifacts due to nonspecific staining, a droplet area of 1 μm^2^ was set as a minimum threshold. The size distribution of droplets was skewed to the left in both strains, with most droplets being in the smallest detectable size range (1–2 μm^2^). In contrast to the increase in neutral lipid content and LD size, we found no differences in immunodetectable apoB-48 or apoB-100, the major structural proteins in VLDL, in liver lysates of KO and WT mice (Fig. 4*A*). Thus, inactivation of *Tm6sf2*^−/−^ led to accumulation of neutral lipids in the liver without changing the steady state levels of apoB-100 or apoB-48.

##### Evidence of Hepatic Injury in Tm6sf2^−/−^ Mice

Genetic variation in *TM6SF2* is associated with steatohepatitis in humans (13, 56). Therefore, we screened the KO mice for early stigmata of hepatic injury. Serum levels of alanine transaminase, a liver enzyme released into the circulation in response to hepatocyte injury, were higher in male KO mice than in WT animals (Fig. 4*B*), although levels of hepatic transcripts encoding genes associated with inflammation and fibrosis and the unfolded protein response were similar in KO and WT animals (Fig. 4*C*). Hematoxylin and eosin staining of liver sections did not reveal pathological stigmata of liver injury; no mononuclear infiltrates, ballooning of hepatocytes, or fibrin deposition was seen in the livers of the KO mice (data not shown).

##### Effects of Tm6sf2 Inactivation on mRNA Levels of Selected Hepatic Lipid Genes

**FIGURE 5.**
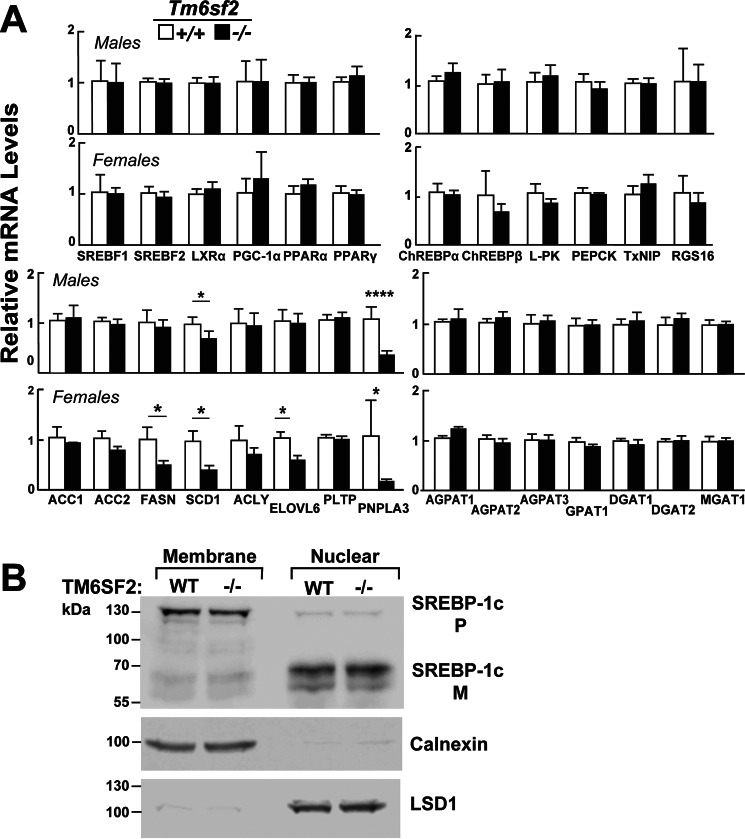
**Relative mRNA levels of selected genes involved in cholesterol and triglyceride metabolism in the livers of WT and *Tm6sf2*^−/−^ mice (*A*) and hepatic SREBP-1c cleavage (*B*).** *A*, quantitative real-time PCR assays were performed to assess the relative levels of selected mRNAs in livers of the 13-week-old chow-fed male mice (5 mice/group) and in 11-week-old chow-fed female mice (6 mice/group) described in the legend to Fig. 3*A*. Expression levels were normalized to levels of 36B4 and expressed relative to levels of WT transcript. Values are means ± S.E. (*error bars*). The official gene symbols were used for all of the genes with the following exceptions: *ATGL* (adipose TG lipase), PNPLA2; *LXR*α, NR1H3; *PGC-1*α, PPARGC1A; *L-PK*, PKLR; *PEPCK*, PCK1; *ChREBP*, MLXIPL. *B*, SREBP-1c regulation in *Tm6sf2* KO mice. Nuclear and membrane fractions were isolated from livers of 18-week-old refed male mice (*n* = 4) by ultracentrifugation as described under “Experimental Procedures.” Lysates from each mouse were pooled, and 40 μg of pooled protein was size-separated on SDS-10% polyacrylamide gels. Proteins were transferred to nitrocellulose membranes and blotted with rabbit anti-mouse mSREBP-1c antibody. The bands were visualized by ECL and quantified using a LI-COR Odyssey Fc imager. The membranes were then stripped and reblotted with antibodies against calnexin and LSD1. The experiment was repeated with 13-week-old females, and the results were similar. *, *p* < 0.05; ****, *p* < 0.0001.

**FIGURE 6.**
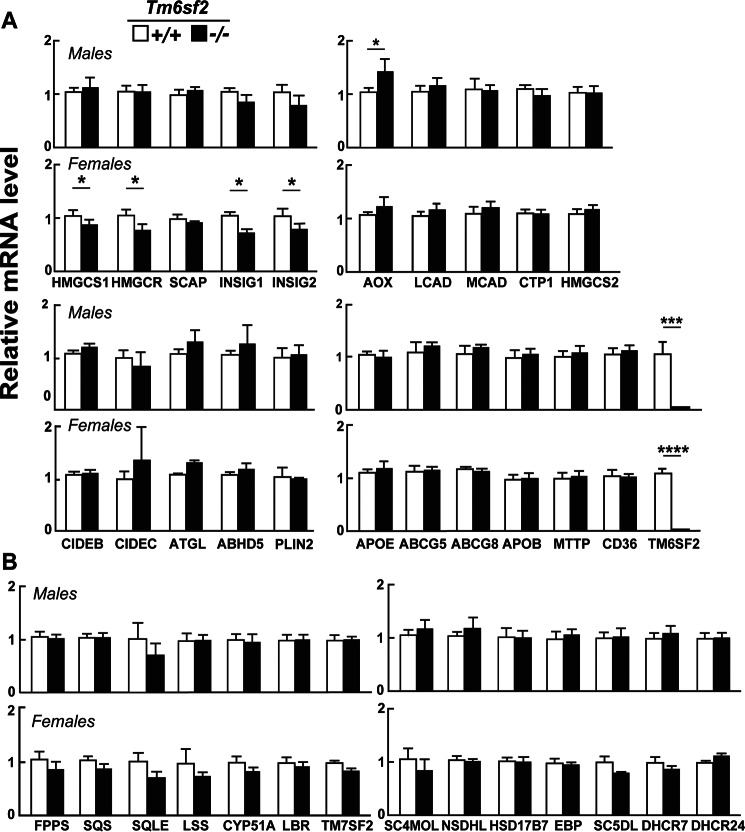
**Relative mRNA levels of selected genes involved in cholesterol metabolism in the livers of WT and *Tm6sf2*^−/−^ mice.** *A* and *B*, quantitative real-time PCR assays were performed to assay the relative levels of selected mRNA levels in livers of the mice described in the legends to Figs. 3*A* and 5*A*. Expression levels were normalized to levels of 36B4 and expressed relative to levels of WT transcript. Values are means ± S.E. (*error bars*). The official gene symbols were used for all the genes with the following exceptions: *AOX*, ACOX1; *MCAD*, ACADM; *FPPS*, FDPS; *SQS*, FDFT1; *SC4MOL*, MSMO1. *HMGCR*, HMG-CoA reductase; *HMGCS1*, HMG-CoA synthase. *, *p* < 0.05; ***, *p* < 0.001; ****, *p* < 0.0001.

Hepatic expression of genes involved in the transcriptional regulation, synthesis, storage, lipolysis, and secretion of lipids and lipoproteins was quantitated by real-time PCR after a 4-h fast in chow-fed mice (Fig. 5*A*). Expression of PNPLA3 was markedly reduced in the KO mice. PNPLA3 is a direct target of SREBP-1c, which is an insulin-responsive transcription factor that up-regulates fatty acid synthesis (30). Levels of mRNA encoding other SREBP-1c target genes were unchanged in males and modestly reduced in female KO mice (Fig. 5*A*). No differences in the hepatic levels of either the precursor or mature form of SREBP-1c were found in livers from male (Fig. 5*B*) or female (data not shown) WT and KO mice. No changes were seen in the SREBP-2 target genes in male KO mice, whereas females had reductions in both HMG-CoA synthase (*HMGCS1*) and HMG-CoA reductase (*HMGCR*) mRNA levels (Fig. 6*A*) but not in the mRNAs encoding other enzymes in the cholesterol biosynthetic pathway (Fig. 6*B*). These findings indicate that the absence of TM6SF2 in chow-fed animals increases hepatic TG and cholesteryl ester content with only modest effects on the transcriptional control of fatty acid and cholesterol metabolism.

##### TG Accumulates in Enterocytes of Tm6sf2^−/−^ Mice

**FIGURE 7.**
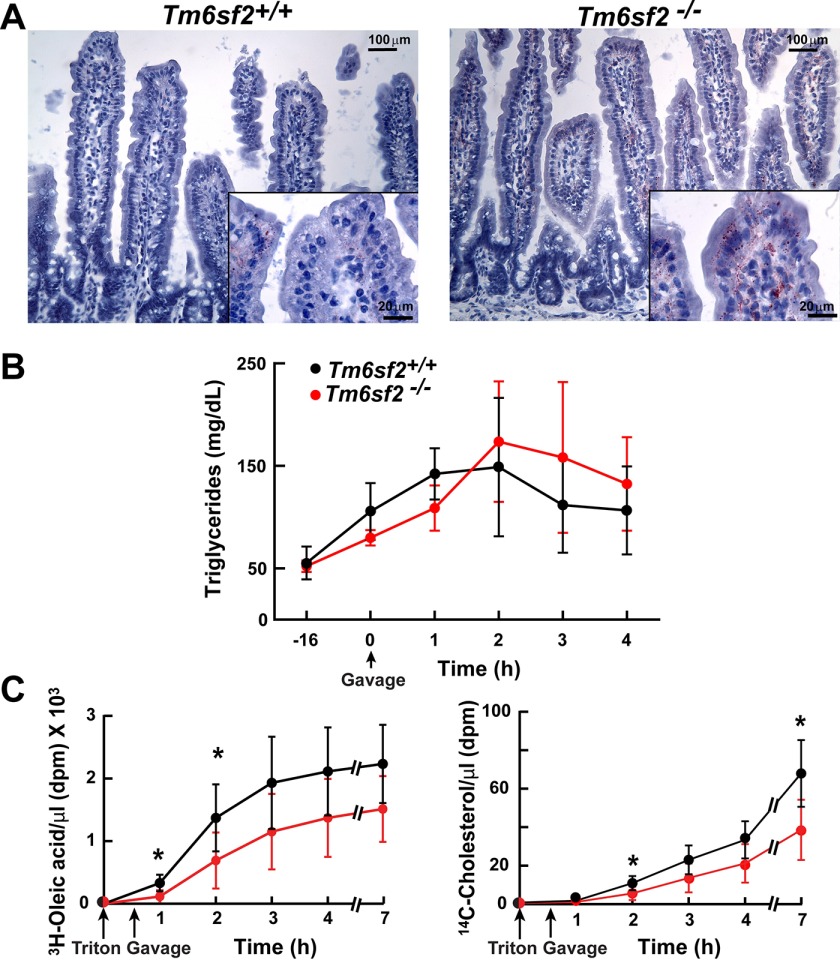
**Intestinal accumulation and absorption of lipid in *Tm6sf2*^−/−^ mice.** *A*, jejunal sections prepared from male mice (18 weeks old) fed a high fat diet for 12 weeks were stained with Oil Red O and visualized using a Leica microscope (DM2000) at ×10 and ×63 (*inset*) magnification as described under “Experimental Procedures.” *B*, chow-fed male mice (*n* = 4 mice/group, 16 weeks old) were fasted for 16 h and then gavaged with corn oil (10 μl/g). Blood was collected at the indicated times after gavage, and plasma levels of TG were measured by enzymatic assay. The experiment was repeated, and the results were similar. *C*, chow fed female mice (*n* = 6 mice/group, 12 weeks old) were fasted for 16 h prior to the injection of Triton WR-1339 (500 mg/kg) into the tail vein. A total of 30 min after the injection, mice were gavaged with corn oil (50 μl) supplemented with 1 μCi of [^14^C]cholesterol (50.8 mCi/mmol) and 5 μCi of ^3^H-labeled oleic acid (54.5 Ci/mmol). Blood was collected at the indicated times before and after injection of Triton WR-1339, and the radioactivity in the plasma was measured by scintillation counting at the indicated time points. *, *p* < 0.05. Values are means ± S.E. (*error bars*).

Like the liver, the intestine is a major site of lipoprotein synthesis. To determine whether TM6SF2 deficiency leads to accumulation of neutral lipids in the intestine, we challenged the KO mice and their WT littermates with a high fat diet (45% lard, 5.5% soybean oil, 17% sucrose, and 19% casein) for 3 months. Oil Red O staining of the jejunum was performed after a 4-h fast. LDs were seen along the basolateral surfaces of enterocytes covering surfaces of the villi but not in the crypts of the KO mice (Fig. 7*A*). Staining was also apparent in the lamina propria of the villi. No lipid accumulation was apparent in enterocytes of WT mice. A similar staining pattern was seen in the duodenum and ileum (data not shown).

##### Absorption of Neutral Lipids Is Modestly but Reproducibly Delayed in Tm6sf2^−/−^ Mice

To determine whether TM6SF2 plays a role in TG absorption, we challenged chow-fed WT and KO mice with an acute bolus of corn oil (10 μl of corn oil/g of body weight) delivered by gavage. Plasma TG levels were measured at the indicated times. The excursion of plasma TG following the gavage was similar in magnitude in WT and KO mice, but peak levels occurred later in the KO mice (1.5 ± 0.5 *versus* 2.7 ± 0.5 h, *p* = 0.0003) (Fig. 7*B*). This finding suggests that TG absorption is preserved but somewhat delayed in KO mice.

To more precisely compare rates of absorption of neutral lipid between the mouse strains, we inhibited lipoprotein lipase by injecting Triton WR-1339 into the tail vein of the mice. We then administered by gavage a solution of corn oil containing ^3^H-labeled oleic acid and ^14^C-labeled cholesterol and measured the appearance of the labeled lipids in the blood. TG and cholesterol absorption were modestly but reproducibly decreased in the *Tm6sf2*^−/−^ mice, particularly at the early time points (Fig. 7*C*).

##### TM6SF2 Is Located in ER and Golgi of Primary Hepatocytes

**FIGURE 8.**
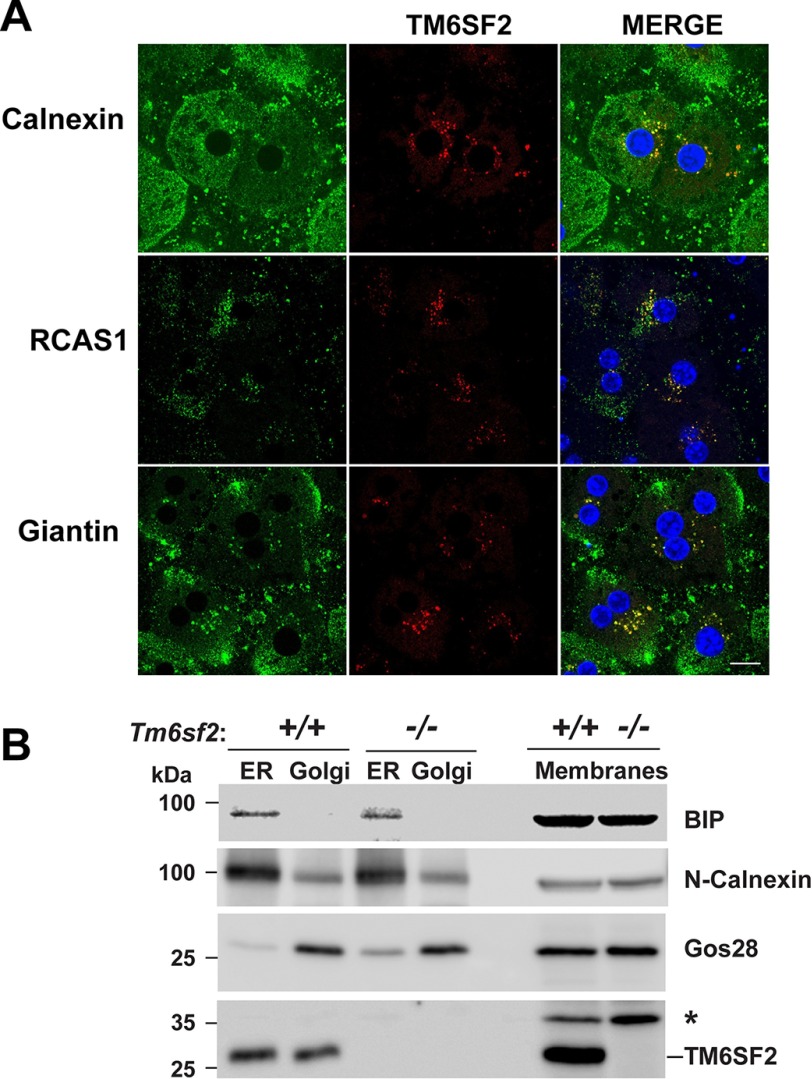
**Subcellular localization of TM6SF2.** *A*, primary hepatocytes from 8-week-old female mice on a chow diet were plated on collagen-coated coverslips for 4 h, fixed, and stained with antibodies against markers for the ER (calnexin (*CANX*)), cis-Golgi (receptor-binding cancer antigen expressed on SiSo cells (*RCAS1*)), and Golgi (Giantin (*GOLGBI*) (*green*, *left column*) and TM6SF2 (*red*, *middle column*)). The merged signal from both channels (*yellow*, *right column*) shows subcellular co-localization. All images were taken using a ×63 oil immersion objective. *Scale bar*, 10 μm. *B*, immunoaffinity isolation of ER and Golgi complex from mouse liver. ER and Golgi fractions were prepared from mouse liver microsomes by immunoaffinity chromatography as described under “Experimental Procedures.” Microsome membranes were dissolved in RIPA buffer, and equal volumes were separated on 10% SDS-PAGE and immunoblotting as described under “Experimental Procedures.” *BiP*, binding immunoglobulin protein; *Gos28*, Golgi SNAP receptor complex member 1; *, nonspecific band.

To define the subcellular location of TM6SF2, we co-stained liver sections of WT mice with antibodies against TM6SF2 and proteins that localize predominantly to ER (calnexin), the cis/medial Golgi (RCAS1), and Golgi matrix (giantin) (Fig. 8*A*). Anti-TM6SF2 antibody showed some degree of co-staining with each marker, indicating that the polytopic protein is present in both the ER and Golgi complex. To confirm these results, we separated mouse liver microsomes by immunoaffinity chromatography on magnetic beads coated with anti-calnexin (ER) and anti-GM130 (Golgi complex) antibodies (Fig. 8*B*). As expected, the immunoisolated ER fraction was enriched in BiP (GRP78, HSPA5) and calnexin and depleted of Golgi SNAP receptor complex member 1 (Gos28, GOSR1), a protein located predominantly in the Golgi complex (31). Reciprocal patterns of expression of these proteins were seen in the Golgi fraction. Immunodetectable TM6SF2 was detected in both the ER and Golgi fractions.

##### Neutral Lipids Accumulate in Cytoplasmic LDs of the Tm6sf2^−/−^ Mice and Do Not Co-localize with TM6SF2

**FIGURE 9.**
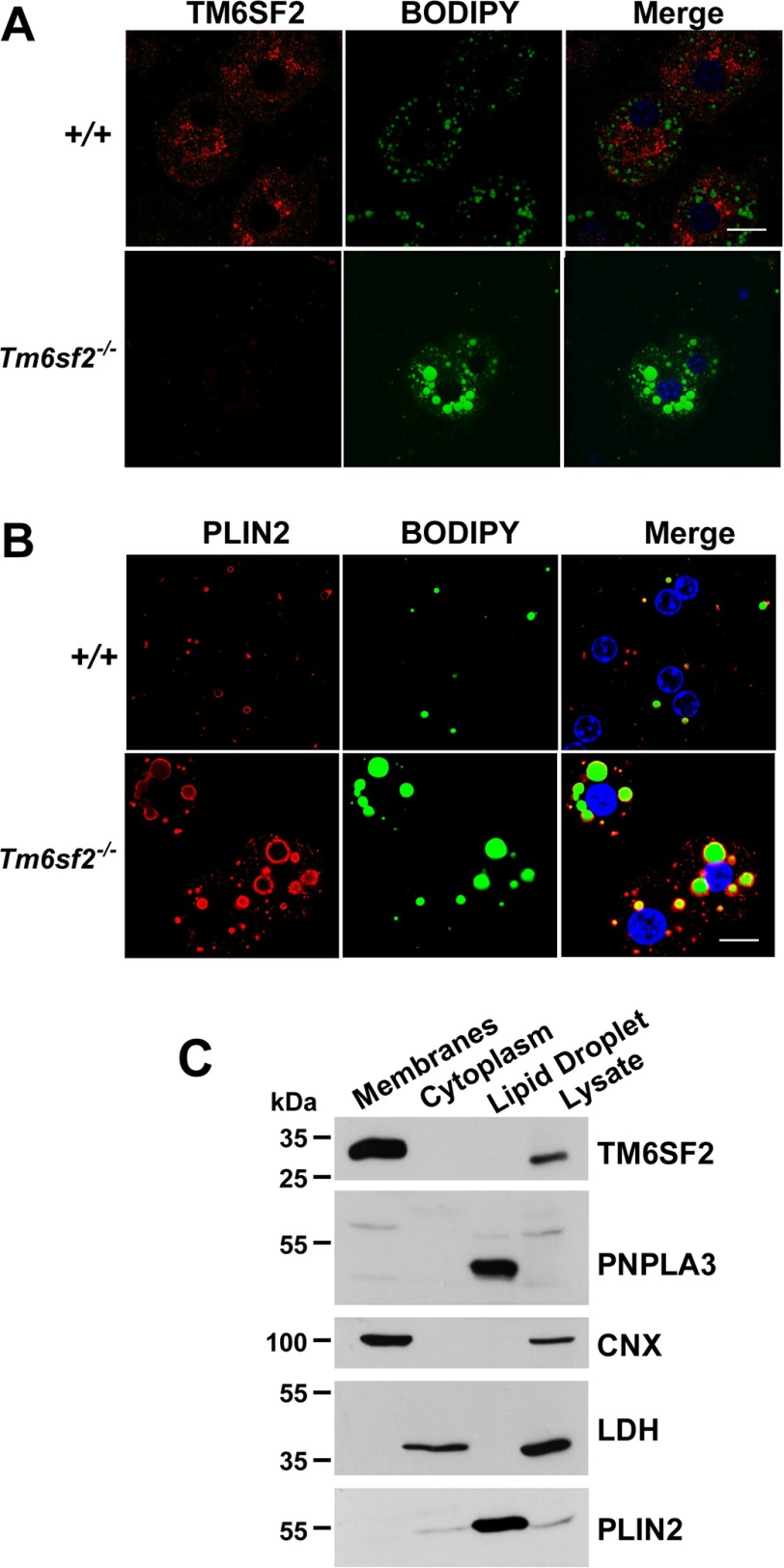
**Localization of TM6SF2 and neutral lipids in mouse primary hepatocytes.** *A* and *B*, primary hepatocytes from 8-week-old female WT mice that were fed chow *ad lib* were plated on coverslips for 4 h, fixed, and stained with BODIPY and with an antibody against TM6SF2 (*A*) and the LD marker PLIN2 (perilipin 2) (*B*). All images were taken using a ×63 oil immersion objective. *Scale bar*, 10 μm. *C*, female C57Bl/N mice (14 weeks old) were fed a high sucrose diet for 2 weeks. Feeding was synchronized for 3 days, and then the mice were killed at the end of the feeding cycle. Livers were homogenized, and the LDs, membranes, and cytosol were separated by ultracentrifugation as described under “Experimental Procedures.” Aliquots of proteins from the membrane and cytosolic fractions (50 μg each) and one-tenth of the LD protein was subjected to 10% SDS-PAGE and immunoblotting as described under “Experimental Procedures.” Calnexin (*CNX*), lactate dehydrogenase (*LDH*), and PLIN2 were used as controls for the ER, cytosolic, and LD fractions, respectively. The experiments were repeated, and the results were similar.

To determine whether the neutral lipid in the liver co-localizes with TM6SF2, we stained primary hepatocytes from WT and KO mice with BODIPY, a neutral lipid dye, and an antibody to TM6SF2. The two signals did not co-localize in WT animals (Fig. 9*A*). We confirmed that the antibody for TM6SF2 was specific because no signal was seen in hepatocytes from the KO animals. To determine whether the lipid that accumulates in the liver was in lipid droplets, we stained sections with a protein marker of LDs, PLIN2 (Fig. 9*B*). The neutral lipid co-localized with the PLIN2 staining. No co-localization of BODIPY staining was seen with markers from the ER (calnexin, CANX) or Golgi complex (giantin, GOLGBI) (data not shown). These findings are consistent with the bulk of neutral lipid in KO mice being located in cytoplasmic LDs.

The exclusion of TM6SF2 from LDs was confirmed by cell fractionation studies. In livers of WT mice fed sucrose to stimulate LD formation, PNPLA3 was detected exclusively in the LD fraction, whereas all immunodetectable TM6SF2 was located in the membrane fraction (Fig. 9*C*). Therefore, unlike PNPLA3, TM6SF2 is not an LD protein.

To determine whether accumulation of lipid in the liver of *Tm6sf2*^−/−^ mice is due to impaired TG secretion, as suggested by our prior experiments (13, 56), we examined and compared plasma lipid and lipoprotein levels in KO and WT mice.

##### Tm6sf2^−/−^ Mice Have Reduced Plasma Levels of Cholesterol but Not ApoB-100 or PCSK9

**FIGURE 10.**
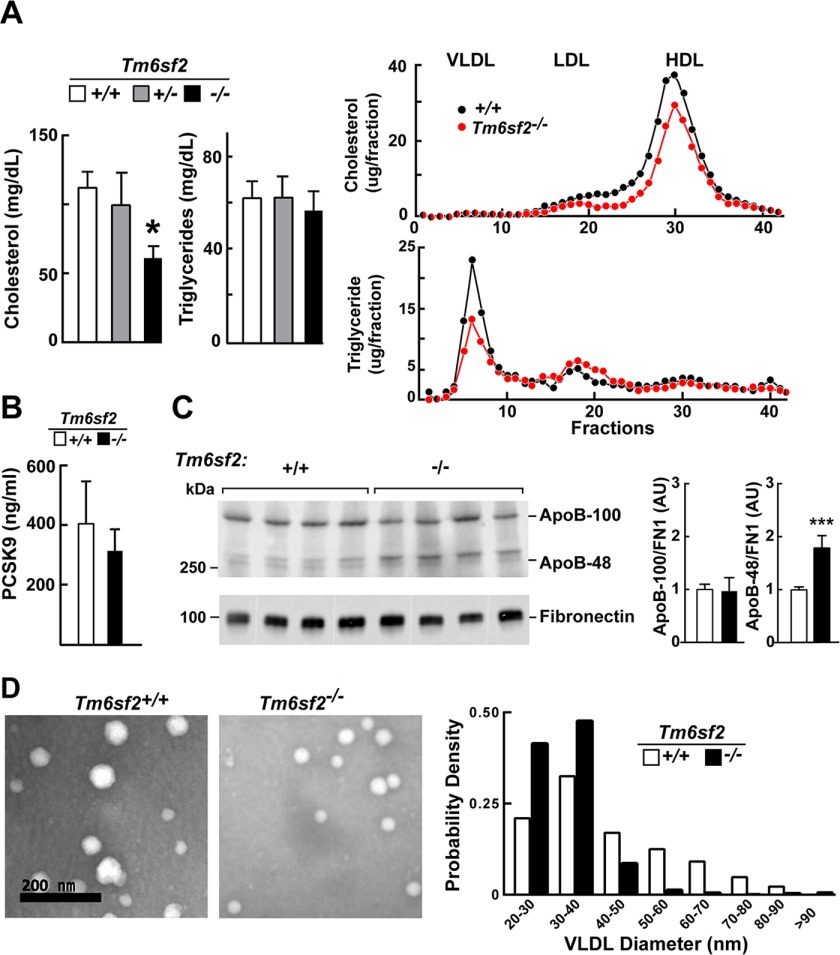
**Plasma levels of lipids, lipoproteins, PCSK9, and apoB in *Tm6sf2*^−/−^ mice.** *A* (*left*), plasma TG and cholesterol levels were measured in chow-fed male WT, *Tm6sf2*^+/−^, and *Tm6sf2*^−/−^ mice (*n* = 5 mice/group, 13 weeks old) using enzymatic assays. *A* (*right*), fast protein liquid chromatography (FPLC) profiles of plasma samples pooled from WT and *Tm6sf2*^−/−^ mice (4 male mice/group, 10 weeks old). Cholesterol (*top*) and TG (*bottom*) were measured in each fraction. Experiments were repeated twice with similar results. *B*, levels of plasma PCSK9 in 11-week-old male chow-fed male mice. Mice (*n* = 5) were metabolically synchronized for 3 days by fasting from 8:00 a.m. to 8:00 p.m. and refed overnight. Blood was collected after the last refeeding period (at 8:00 a.m.), and the plasma levels of PCSK9 were detected using an ELISA as described under “Experimental Procedures.” *C*, plasma (0.2 μl) was size-fractionated on a 4–12% gradient SDS-polyacrylamide gel, and levels of apoB-48 and apoB-100 were determined by immunoblotting analysis using a rabbit polyclonal antibody (Abcam, ab20737; 1:1,000) The signal was detected and quantified using a LI-COR Odyssey Fc imager. Fibronectin was used as a loading control. Values are means ± S.E. (*error bars*). *D*, VLDL particles from plasma of WT and KO female mice (*n* = 3, 20 weeks old) were visualized by electron microscopy as described under “Experimental Procedures.” The size distribution of VLDL particles in 10 randomly selected images was analyzed and compared using ImageJ software as described under “Experimental Procedures.” *, *p* < 0.05; ***, *p* < 0.001.

Previously, we showed that inactivation of TM6SF2 in the liver using shRNAs was associated with a significant reduction in plasma levels of cholesterol and TG (13, 56). In the *Tm6sf2*^−/−^ mice, plasma levels of cholesterol were reduced, reflecting a reduction in cholesterol in both the LDL and HDL fractions (Fig. 10*A*). Despite the reduction in cholesterol, circulating levels of apoE, a ligand for both the LDLR and LDLR-related protein (32), and of apoA1 and ABCA1, proteins required for HDL production (33), were similar in the two strains (data not shown). No differences were found in LDLR and SCARB1 (SR-B1), the major receptors that mediate clearance of LDL or HDL, respectively (data not shown). In addition, circulating levels of PCSK9, a protein that regulates expression of hepatic LDLR (34), the major clearance pathway for apoB-containing lipoproteins, were similar in the two groups of mice (Fig. 10*B*).

*Tm6sf2*^−/−^ mice had less TG in the VLDL fraction but higher TG levels in the LDL fraction compared with WT mice (Fig. 10*A*, *right*). Despite the reduction in VLDL-TG, the levels of apoB-100 were similar in the KO and WT animals (Fig. 10*C*). Surprisingly, the levels of apoB-48 were significantly and reproducibly higher in plasma of *Tm6sf2*^−/−^ mice (Fig. 10*C*). The reason for the increase in apoB-48 levels in the KO mice is not known.

##### Smaller VLDL Particles in the KO Mice

The decrease in VLDL-TG relative to plasma apoB-100 levels implies a reduction in VLDL particle size in the *Tm6sf2*^−/−^ mice. To directly assess VLDL particle size, VLDLs were isolated by ultracentrifugation and visualized by electron microscopy using negative staining (Fig. 10*D*, *left*). The sizes of the VLDL particles varied over a wide range with strong leftward skewing in WT and in KO mice (Fig. 10*D*, *right*). Median particle diameter was smaller in KO than in WT animals (31 *versus* 38 nm, *p* = 1.3 × 10^−86^). KO animals had more small particles (20–40 nm) and very few large particles (>50 nm) relative to WT mice.

The observation that KO mice have smaller VLDLs with reduced TG content but normal levels of apoB-100 and apoB-48 is consistent with a model in which the liver (and possibly also the intestine) secretes apoB-containing lipoproteins that are not fully lipidated. Alternatively, the clearance of large TG-rich lipoproteins may be selectively enhanced in the KO mice.

##### VLDL-TG Secretion Is Reduced but ApoB Secretion Is Preserved in Tm6sf2^−/−^ Mice

**FIGURE 11.**
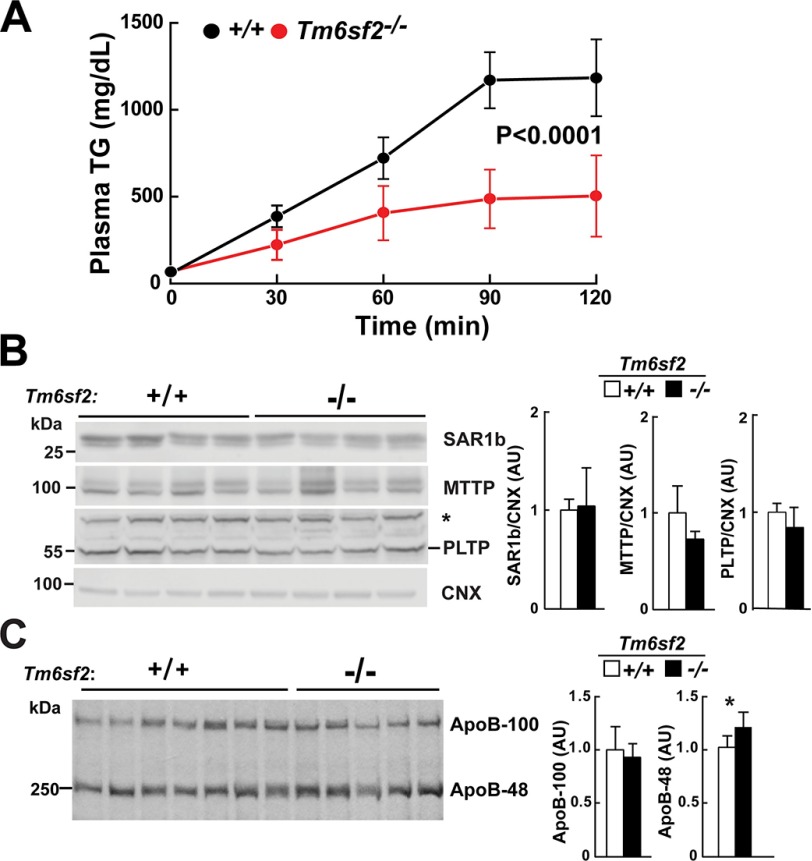
**VLDL secretion in WT and *Tm6sf2*^−/−^ mice.** *A*, VLDL-TG secretion. Triton WR-1339 (500 μg/g) was injected into the tail veins of chow-fed female mice (*n* = 4 mice/group, 9 weeks old) after 4-h fasting. Blood was collected at the indicated time points, and plasma TG levels were measured. Mean TG levels at each point are shown. Rates of VLDL-TG secretion were determined by least squares regression of plasma TG levels plotted against time. Slope estimates were determined from the linear portion of each graph and compared using Student's *t* test. *B*, immunoblotting analysis of SAR1b (secretion-associated, Ras-related GTPase 1B), microsomal TG transfer protein (*MTTP*), and phospholipid transfer protein (*PLTP*). Liver lysates (40 μg) were size-fractionated by 10% SDS-PAGE and then incubated with primary and secondary antibodies, as described under “Experimental Procedures.” *, nonspecific band. *C*, [^35^S]methionine incorporation in apoB. Male mice (*n* = 5, age = 9 weeks) were synchronized on a 3-day regimen of fasting (8:00 a.m. to 8:00 p.m.) and refeeding (8:00 p.m. to 8:00 a.m.). At 8:00 a.m. on day 4, food was withdrawn, and mice were fasted for 4 h and then injected via the tail vein with 200 μCi of [^35^S]methionine (1,175 Ci/mmol) and Triton WR-1339 (500 mg/kg). Blood was collected from the tail vein before and 45 and 90 min after the injection. Plasma samples from the 90 min point were treated with deoxycholate/trichloroacetic acid to precipitate proteins. The pellets were washed with acetone and dissolved in 200 μl of 2% SDS, 1 m urea buffer. Loading buffer (50 μl of 5× Western blot loading buffer) was added, and 20-μl aliquots were size-fractionated by 5% SDS-PAGE. Gels were dried and exposed to x-ray film (BIOMAX XAR) for 4 days at −80°C. Band intensity was quantified using the LI-COR Image Studio software. The experiment was repeated, and the results were similar. *, *p* < 0.05. *AU*, arbitrary units.

To establish whether the low VLDL-TG in the KO mice was due to reduced VLDL-TG synthesis or to accelerated VLDL-TG clearance, we inhibited lipoprotein lipase using Triton WR-1339 (500 mg/kg) and monitored the appearance of TG in the circulation (Fig. 11*A*). The rate of TG secretion (determined from the slope of plasma TG levels plotted against time) was markedly reduced in KO animals when compared with WT littermates (225 ± 102 *versus* 600 ± 95 mg/dl/h, *p* < 0.0001).

To determine whether the reduction in VLDL-TG accumulation was due to a defect in VLDL particle synthesis or secretion, we examined the levels of key proteins involved in these processes. Chylomicron and VLDL synthesis are both severely compromised in two recessive disorders causing severe hypocholesterolemia: chylomicron retention disease, which is caused by Sar1b deficiency (35), and abetaliproteinemia, which is due to deficiency of MTTP (36). Levels of both SAR1B and MTTP did not differ between WT and KO mice (Fig. 11*B*). Synthesis of apoB-100-containing lipoproteins is also compromised in mice lacking phospholipid transfer protein, the enzyme that transfers phospholipids onto the nascent VLDL particle (37). No differences in levels of phospholipid transfer proteins were found in the KO and WT mice (Fig. 11*B*).

To determine whether apoB secretion was decreased in a similar manner to VLDL-TG, which would be consistent with a defect in whole particle secretion, we injected mice with [^35^S]methionine and Triton WR-1339 and measured the incorporation of radiolabel into circulating apoB-48 and apoB-100 after 90 min. The accumulation of label in apoB-100 was similar in WT and KO mice (Fig. 11*C*). This finding, together with the similar levels of apoB-100 in both the liver (Fig. 4*A*) and the plasma (Fig. 10*C*), is consistent with similar rates of apoB-100 secretion in WT and KO mice. Thus, TM6SF2 absence does not compromise the secretion of VLDL particles; rather, it reduces the lipidation of each particle. Interestingly, accumulation of label in apoB-48 was greater in KO than in WT animals and comparable with the increase in plasma levels of apoB-48 (Fig. 10*C*). In liver-specific *Mttp*^−/−^ mice, no apoB-100 is detected in the plasma, but levels of apoB-48 are similar to those in WT animals (38), perhaps because lipoproteins containing apoB-48 require less lipidation for secretion.

These data support the hypothesis that livers of *Tm6sf2*^−/−^ mice secrete normal numbers of poorly lipidated lipoproteins. This finding suggests that TM6SF2 plays a role in the bulk lipidation step that leads to maturation of VLDL particles in the liver.

##### Modest Alterations in Fatty Acid Composition in TG and Phosphatidylcholine in Tm6sf2^−/−^ Mice

**FIGURE 12.**
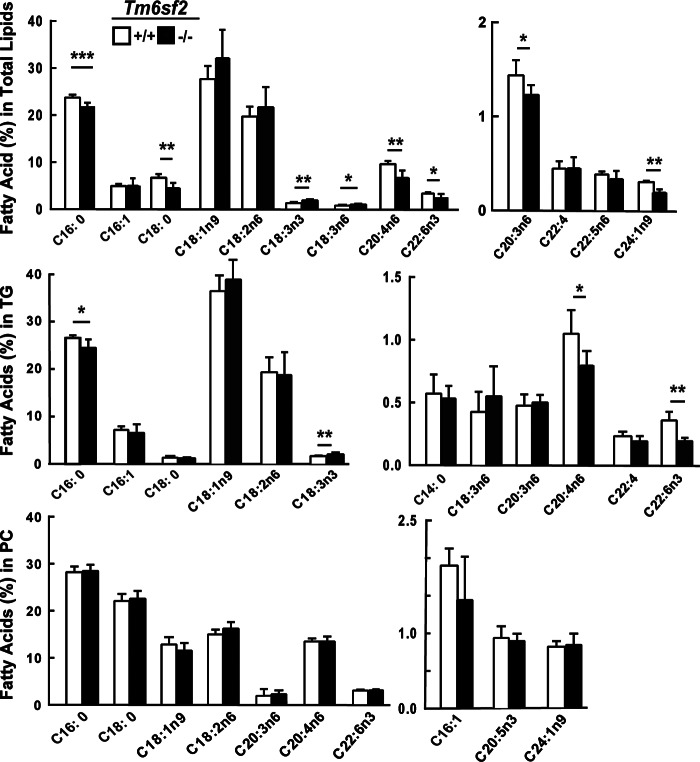
**Fatty acid composition of hepatic lipids in WT and *Tm6sf2*^−/−^ mice.** Livers were collected from chow-fed 13-week-old male mice (*n* = 5) after a 4-h fast. The TG and PC fractions were separated by thin layer chromatography (*TLC*), hydrolyzed, and derivatized with trimethylsilane. The fatty acid methyl-esters were quantified by GC. Each value represents the mean ± S.E. (*error bars*). *, *p* < 0.05; **, *p* < 0.01; ***, *p* < 0.001.

Mice lacking the lysophosphatidylcholine acyltransferase LPCAT3 have several features in common with *Tm6sf2*^−/−^ mice, including hepatosteatosis, reduced VLDL-TG secretion, smaller VLDL particles (23, 39), and accumulation of lipid in enterocytes (40). Genetic disruption of *Lpcat3* sharply reduces levels of arachidonic acid-containing phosphatidylcholines in liver and circulating VLDL. Thus, we measured the distribution of fatty acids in lipids extracted from livers and plasma of KO and WT mice. Although some small differences in fatty acid composition of phospholipids were observed in WT and KO mice, the arachidonic acid content of the phospholipid fraction was remarkably similar in the two groups (Fig. 12).

## Discussion

**FIGURE 13.**
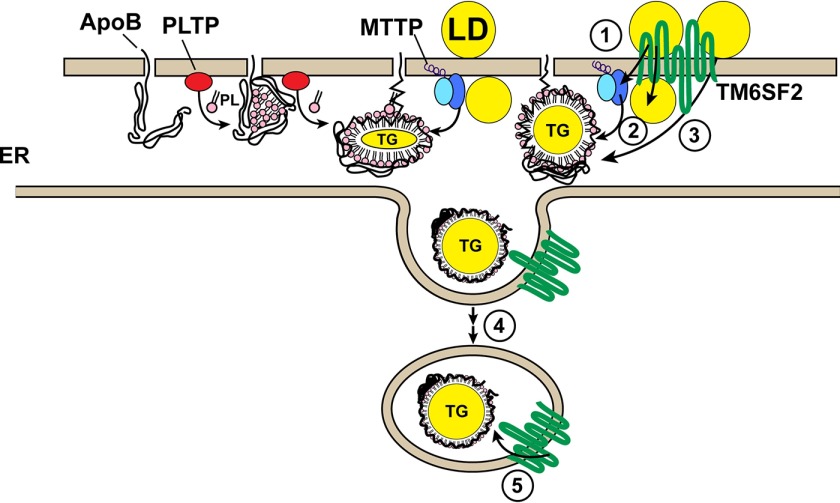
**Schematic illustration of the role of the TM6SF2 in VLDL lipidation.** VLDL synthesis is initiated in the ER with the co-translational addition of phospholipids to apoB. The addition of TG to the particle also begins in the ER, a process that requires MTTP. The partially lipidated VLDL particle is packaged into COPII vesicles and exported to the Golgi, where they appear to undergo further “bulk phase” lipidation. Our present data are most consistent with a model in which TM6SF2 promotes this bulk phase lipidation, either by transporting neutral lipids from lipid droplets to the particle by transferring lipid to MTTP (➀), to neutral LD in the ER lumen (➁), or directly to the nascent VLDL particle (➂). Alternatively, TM6SF2 could participate in the transfer of lipid to the particle en route to or within the Golgi complex (➃ and ➄).

A major finding of this study is that the absence of TM6SF2 causes hepatic steatosis and elevated alanine transaminase levels in the absence of a dietary challenge. We provide evidence that TM6SF2 is required for normal lipidation of TG-rich lipoproteins in the liver and possibly in the intestine, but is not required for secretion of apoB-containing lipoproteins. We show that TM6SF2, which is predicted to have 10 transmembrane helices, is located in both the ER and the Golgi complex, whereas the lipid that accumulates in the livers of mice expressing no TM6SF2 is located in LDs. The large decrease in TG secretion in KO mice (Fig. 11*A*) indicates that TM6SF2 plays a role in VLDL assembly (Fig. 13). We found no evidence for impaired secretion of apoB-100 and apoB-48 into the circulation (Fig. 10*C*). Thus, TM6SF2 is not required for the initial lipidation of apoB, for the packaging of VLDL into COPII vesicles to exit the ER, or for secretion of VLDL particles from cells. Rather, the decreased size and TG/APOB ratio in circulating TG-rich lipoproteins of KO mice (Fig. 10*D*) are consistent with the hypothesis that these mice secrete normal numbers of VLDL particles that are relatively lipid-poor. Taken together, our results indicate that TM6SF2 promotes the addition of neutral lipid to nascent apoB-containing lipoproteins in hepatocytes and also possibly in enterocytes. The possible functional roles of TM6SF2 are summarized in the legend to Fig. 13.

Despite a striking reduction in VLDL-TG secretion, mice lacking TM6SF2 had no apparent defect in energy metabolism or in the metabolism of free fatty acids in the liver. This finding is consistent with our prior observation that body weight and insulin sensitivity are not altered in humans with loss-of-function mutations in TM6SF2 (13, 56). The observation that hepatic expression of TM6SF2 was not affected by nutritional status (Fig. 1*C*, *right*), together with the finding that hepatic expression of genes involved in fatty acid metabolism are modestly altered in the KO mice (Fig. 5*A*), is consistent with the hypothesis that TM6SF2 plays a constitutive role in efflux of hepatic fatty acids in the form of VLDL-TG rather than in the biosynthesis or oxidation of fatty acids in hepatocytes.

The only gene whose expression was markedly affected by disruption of *Tm6sf2* was *Pnpla3* (Fig. 5*A*), a LD protein that we previously found to be associated with liver fat content (5). Deletion of PNPLA3 in mice does not lead to accumulation of liver fat (41, 42); therefore, the low PNPLA3 transcript levels in TM6SF2 KO mice are likely to be a consequence, rather than a cause, of the increase in hepatic TG in these animals. PNPLA3 is an SREBP-1c target gene (30), and it is possible that the PNPLA3 gene is sensitive to small changes in SREBP-1c activity. Alternatively, PNPLA3 may be subject to regulation by other, as yet unidentified factors that are altered in the *Tm6sf2* KO mice.

The specific biochemical action of TM6SF2 in VLDL remains to be defined. Mutations in proteins that mediate the early lipidation (36, 43) and COPII packaging (44) of VLDL markedly reduce secretion of apoB. Because apoB secretion was not decreased in *Tm6sf2* KO mice, it is unlikely that TM6SF2 is required for any of the early steps in VLDL assembly. VLDL particles are further enriched with TG as they traverse the ER/Golgi secretory pathway (45). The mechanism by which additional TG molecules are added to particles has not been fully defined. One hypothesis is that the lipid-poor VLDL fuses with LDs in the microsomal lumen (45). Ultrastructural studies of hepatocytes from liver-specific *Mttp* KO mice reveal a key role for MTTP in the formation of large LDs in the microsomal lumen (38), but incorporation of TG from lumenal droplets into nascent VLDL does not require MTTP (46). It is tempting to speculate that TM6SF2 acts downstream of MTTP to facilitate bulk lipidation of the nascent VLDL particle, by transferring neutral lipids either from cytoplasmic or from intraluminal LDs to nascent VLDL particles (Fig. 13). Alternatively, TM6SF2 may have an enzymatic activity, as suggested by a recent study showing that TM6SF2 shares a domain with the cholesterol biosynthetic enzyme EBP (Δ8,Δ7-sterol isomerase) (47).

Despite markedly reduced production of VLDL-TG in the *Tm6sf2* KO mice (Fig. 10*A)*, plasma levels of TG in these animals were similar to those of their WT littermates. This result differs from our previous finding that inactivation of *Tm6sf2* in mouse liver using shRNA decreases plasma levels of TG (13, 56). The reason for the different effects of TM6SF2 inactivation on plasma TG levels in these two mouse models is not clear at this time. It is possible that the shRNAs that we used to knock down TM6SF2 have off-target effects in the liver. Alternatively, chronic inactivation of TM6SF2 may result in compensatory changes in TG metabolism that mitigate changes in plasma TG levels. Because VLDL-TG secretion is reduced in the *Tm6sf2*^−/−^ mice, the normal TG levels in these mice imply a decreased rate of clearance of TG. Fisher *et al.* (48) showed that large VLDLs are much better substrates for lipoprotein lipase, the enzyme that mediates hydrolysis of VLDL-TG. The VLDL particles in the *Tm6sf2* KO mice are smaller than those of WT mice and may be hydrolyzed more slowly and thus remain longer in the circulation than do VLDL from WT mice. It is also possible that rapid initial hydrolysis of VLDL-TG in the KO mice leads to redistribution of TG to LDLs, which are then cleared more slowly from the circulation.

We also observed accumulation of neutral lipids in the intestine, the other major site of expression of TM6SF2, and of synthesis/secretion of apoB-containing lipoproteins. For reasons that are not clear, neutral lipids accumulate in the stroma of villus as well as in the absorptive cells of the small intestine (Fig. 7*A*). It is interesting that TM6SF2 expression is high in all three regions of the small intestine, in contrast to other proteins involved in lipid absorption and chylomicron synthesis, such as NPC1L1, MTTP, and Sar1b (49–51). TM6SF2 may have a function independent of its role in the formation of apoB-containing lipoproteins. Additional studies will be required to fully characterize the role of TM6SF2 in the intestine.

The most striking difference in the plasma lipid levels of KO and WT mice was in the level of cholesterol (Fig. 10*A*). This pattern mirrors the effect of the TM6SF2-E167K variant observed in humans (13, 56). The changes in plasma cholesterol levels in *Tm6sf2*^−/−^ mice were not accompanied by any major changes in the hepatic expression of genes encoding proteins involved in cholesterol synthesis (Fig. 6) or of the major receptor that removes LDL particles from the plasma, LDLR (data not shown). The reduction in HDL-C without a concomitant reduction in apoA1 (data not shown) would be consistent with an increase in activity of SR-BI, which selectively removes cholesterol esters from circulating lipoproteins (52). However, no differences were detected in SR-BI levels in KO animals when compared with WT littermates (data not shown). Mice lacking MTTP in the liver also have low plasma HDL-C levels, presumably due to reduced availability of VLDL lipids for transfer to HDL (38). The low HDL-C in *Tm6sf2*^−/−^ mice may be due to decreased secretion of VLDL lipids in these animals.

The reciprocal effects of TM6SF2 inactivation on the accumulation of TG in cytoplasmic LDs and the secretion of VLDL-TG by the liver suggests that the two processes are tightly coupled. This finding has significant implications for current models of LD function. LDs are usually viewed as organelles for localized energy storage. However, under physiological conditions, hepatocytes of WT mice secrete large amounts of TG (6 mg/h in the present study assuming a plasma volume of 1 ml). Given a hepatic TG content of 10–20 mg, the turnover of TG in cytoplasmic LDs is rapid. Hepatocytes only accumulate TG when an influx of fatty acids exceeds the capacity of cells to package and secrete TG in VLDL. When the influx of fatty acids is reduced, liver TG content decreases rapidly (53). These findings suggest that in contrast to the large unilocular LDs in adipocytes, which are adapted for long term, high volume TG storage, the small multilocular LDs in hepatocytes function to rapidly but transiently buffer free fatty acids en route to oxidation in mitochondria or secretion in VLDL-TG.

Continued loading of hepatocytes with fatty acids, as occurs in obesity, elicits a maladaptive inflammatory response that can progress to fibrosis and, ultimately, cirrhosis (54). The increase in serum alanine transaminase levels in *Tm6sf2*^−/−^ mice suggests that hepatic steatosis in these animals is associated with injury. This finding contrasts with models of PNPLA3-I148M-associated steatosis, which have increased levels of hepatic fat but not serum enzymes (27, 55). The reason for this difference is not clear. The steatosis and transaminitis in *Tm6sf2*^−/−^ mice were observed on a standard chow diet. In contrast, the PNPLA3-I148M knock-in mice did not develop steatosis unless they were placed on a high sucrose diet, which causes an increase in hepatic fatty acid synthesis and PNPLA3 levels (27). The *Tm6sf2*^−/−^ mice may provide an improved model for the progression of fatty liver disease. Further studies will be required to determine whether dietary manipulation can elicit chronic liver disease in these animals.

## Author Contributions

E. S. conducted most of the experiments, analyzed the results, and wrote the paper. S. G. conducted some of the experiments and analyzed the results. S. B. R. conducted all imaging studies. J. C. and H. H. H. conceived the idea for the project, analyzed and interpreted data, and wrote the paper. All authors reviewed the results and approved the final version of the manuscript.

## Footnotes

4 The abbreviations used are:

NAFLD: nonalcoholic fatty liver disease
ER: endoplasmic reticulum
LD: lipid droplet
TG: triglyceride
HSD: high sucrose diet
RIPA: radioimmune precipitation assay
BisTris: 2-[bis(2-hydroxyethyl)amino]-2-(hydroxymethyl)propane-1,3-diol
Tricine: *N*-[2-hydroxy-1,1-bis(hydroxymethyl)ethyl]glycine
MTTP: microsomal triglyceride transfer protein.

## Note Added in Proof

Fan *et al.* (57) has generated a mouse in which *Tm6sf2* was disrupted using CRISPR-Cas9. In contrast to our mice, these KO mice did not have hepatic steatosis. The reason for the discrepancy between these two models is not clear.
